# Using Observational Dyadic Methods in Youth Mentoring Research: Preliminary Evidence of the Role of Actors’ and Partners’ Self-disclosure in Predicting Relationship Quality

**DOI:** 10.1007/s10964-023-01757-y

**Published:** 2023-03-04

**Authors:** Hilary Dutton, Kelsey L. Deane, Nickola C. Overall

**Affiliations:** 1 Faculty of Education, University of Canterbury, Christchurch, New Zealand; 2 Faculty of Education and Social Work, University of Auckland, Auckland, New Zealand; 3 School of Psychology, University of Auckland, Auckland, New Zealand

**Keywords:** Youth mentoring, Self-disclosure, Relationship quality, Behavioral observation, Actor partner interdependence model

## Abstract

Self-disclosure builds high quality relationships, but knowledge of self-disclosure in youth mentoring relationships is limited by a lack of research and reliance on self-reports. To demonstrate the value of observational methods and dyadic modeling of mentoring communication processes, this study examined the associations between behavioral observation of self-disclosure and self-reported relationship quality in 49 mentee-mentor dyads (mentees: 73.5% female; x̄ age = 16.2, range = 12–19; mentors: 69.4% female; x̄ age = 36.2, range = 19–59). Video-recorded observations of disclosure were coded on three dimensions: amount (number of topics and detail of disclosure), intimacy (disclosure of personal or sensitive information), and openness (willingness to disclose). More intimate mentor disclosure was associated with higher mentee relationship quality, whereas higher amount of mentor disclosure combined with low intimacy was associated with lower mentee relationship quality. Greater mentee openness correlated with higher mentor relationship quality, but more intimate mentee disclosures were associated with lower mentee relationship quality. These preliminary findings illustrate the potential of methods that enable in-depth investigation of dyadic processes to advance understanding of how behavioral processes may influence mentoring relationships.

## Introduction

The effectiveness of formal youth mentoring programs in producing positive developmental outcomes for adolescents is largely contingent on mentors and mentees building and sustaining quality relationships (Dutton et al., 2022). Higher relationship quality is associated with better mentee outcomes, such as academic outcomes (Bayer et al., 2015), improved relationships (Chan et al., 2013), and prosocial behavior (Deutsch et al., 2013). A growing body of research on relationship processes in youth mentoring has enhanced understanding of the features of, and factors that contribute to, effective mentoring relationships, but the existing research is limited by retrospective, self-reported and globalized accounts that restrict understanding of how dyadic processes influence relationship outcomes (Pryce et al., 2021). To illustrate how greater use of methods that assess dyadic behavioral processes across mentors and mentees could advance understanding of mentoring relationships, this study used behavioral observations of self-disclosure and Actor-Partner Interdependence Modeling to examine what type of mentor and mentee self-disclosure is associated with relationship quality for both mentors and mentees. In doing so, this article offers some preliminary evidence and insights into how behavioral observations and dyadic analytic methods enable investigation of communication processes in mentoring relationships beyond the limitations of self-report questionnaire studies.

### Self-disclosure and Relationship Quality: The Importance of Behavioral and Dyadic Methods

Mentoring relationships develop, falter, or thrive depending on dyadic communication behaviors. Yet the influence of these complex processes remains obscured, in part because the methods commonly applied in this field—notably self-report questionnaires—are not suited to investigating them. Behavioral observational methods offer substantial benefits by capturing processes within relationship interactions in real-time and within the dyadic context. Self-disclosure is a pivotal example of such a process. Decades of scholarship show that repeated engagement in disclosure facilitates positive feelings about relationships, such as closeness, trust, and satisfaction (Willems et al., 2020). Disclosure also helps sustain relationships over time by signaling commitment and care for the relationship (Tardy & Smithson 2018). Individuals in a relationship move between the roles of discloser and receiving partner within and across conversations during the course of their relationship (Greene et al., 2006). Thus, disclosure is a dyadic, interdependent process in which both parties’ disclosure will contribute to relationship quality.

Consistent with the wider literature, recent evidence indicates that greater mentor disclosure is associated with higher mentee relationship quality (Dutton et al., 2022). However, self-reports provide limited insight into behavioral processes, like disclosure, that occur within dyadic interactions. Self-reports are susceptible to biases based on participants’ own interpretations and individualized recollections of past interactions. For example, participants are more likely to report recent events and may struggle to accurately recall behaviors they express within specific interactions (Pryce et al., 2021). Behavioral observation not only limits these biases by enabling researchers to apply standardized assessments, but also offers benefits in examining multidimensional constructs. Specific behaviors can be isolated during analysis that helps pinpoint effects that are associated with specific dimensions. For instance, the intimacy (disclosure of personal or sensitive information) of disclosure has been established as more critical for relationship development than the overall amount (number of topics and detail of disclosure) of disclosure (Altman & Taylor, 1973).

Behavioral methods are also critical because they capture the dyadic nature of disclosure processes. As described above, mentors and mentees move between being the discloser (actor) or the receiver (partner), shaping the relationship quality of the other. Disclosure scholarship emphasizes the importance of *actors’* self-disclosure in facilitating receiving *partners’* feelings of trust, closeness, and relationship quality (Willems et al., 2020). In the mentoring context, examining these partner effects requires measuring the self-disclosure and relationship outcomes of both mentors and mentees, and then applying dyadic modeling to assess, for example, the associations between mentors’ disclosure and mentees’ relationship quality while accounting for the role of mentees’ disclosure (and vice versa). This captures the inherently dyadic nature of communication processes, and more fully evaluates the various ways mentors’ and mentee’s self-disclosure may differentially shape relationship quality. For instance, the different role expectations and power dynamics across mentors and mentees (Keller & Pryce, 2010) may mean that mentee disclosure could have positive associations with mentors’ relationship quality (positive partner effect), but leave mentees feeling vulnerable (negative actor effect). These important associations can only be uncovered by examining dyadic processes within mentor-mentee disclosure interactions using appropriate methods.

**Fig. 1:**
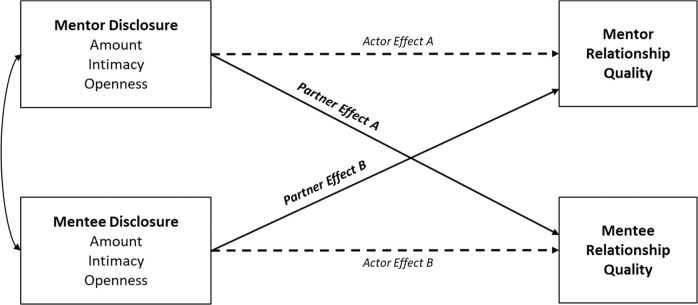
Actor and partner effects of amount, intimacy and openness of disclosure on mentees’ and mentors’ relationship quality

The current study offers a proof-of-concept illustration of the value of observational methods and dyadic modeling of mentoring communication processes. The benefits behavioral observation methods offer—including bypassing self-report biases, differentiating between theoretically-relevant behavioral dimensions, assessing how both mentors and mentees behavior shapes the outcomes of each other—have led to calls for their increased use in youth mentoring research (Deutsch & Spencer, 2009) as well as in self-disclosure research more generally (Dindia et al., 2002). The aim of this study is to show how employing behavioral methods better suited for analyzing dyadic processes in mentoring relationships could make a significant contribution to understanding relational processes like self-disclosure. To illustrate, Fig. 1 outlines the possible actor and partner effects of self-disclosure observed within mentor-mentee interactions on both mentors’ and mentees’ relationship quality. This actor-partner model recognizes that both mentors’ and mentees’ disclosures as actors are likely to be associated with their partners’ evaluations of the quality of their mentoring relationships (partner effects shown by the solid lines) and may also be associated with their own evaluations of relationship quality (actor effects shown by the dashed lines). Moreover, both the partner and actor effects may differ across mentor and mentee roles. Finally, the partner and actor effects for both mentors and mentees may differ according to the dimensions of disclosure. The following sections describe the anticipated partner effects of mentor, then mentee, disclosure focusing on three key dimensions: amount, intimacy, and openness. The possible actor effects of mentee and mentor disclosure are also outlined.

#### Partner effects of mentors disclosing to mentees

The emerging research on disclosure in the mentoring context focuses on mentors as actors disclosing to mentees as social partners (see Partner Effect A in Fig. 1). Given the purpose and expectation of mentoring to support youth development, the impact of relational processes on mentees is of particular concern, and thus outcomes of interest are usually associated with mentee development and relationship experiences. Theoretically, mentors’ disclosure may have positive effects on mentees’ relationship evaluations through the development of closeness and trust. However, questionnaire-based measures of disclosure in mentoring have not measured specific dimensions of disclosure—namely amount, intimacy, and openness—that theory and research outside the context of mentoring suggests is important (Altman & Taylor, 1973). It is therefore unknown whether any specific dimensions of disclosure make unique contributions to relationship quality.

Disclosure amount includes the number of topics discussed and how much information or detail is shared. A higher versus lower amount of disclosure implies closer, more trusting relationships (Altman & Taylor, 1973), whereas actors may purposefully reduce or withdraw disclosure when a relationship is deteriorating (Willems et al., 2020). Compared to other types of close relationships, mentor-mentee dyads may be less likely to engage in equitable amounts of disclosure due to the focus on mentee development. Mentors may disclose less, or be expected to disclose less, to give space to mentees and avoid dominating interactions. High amounts of mentor disclosure, therefore, might risk undermining mentees’ views of the mentoring relationship.

Intimacy of disclosure describes actors progressively disclosing more personal, sensitive information that reveals more of the self to a partner. Actor’s increasingly intimate disclosures invite the partner to disclose in a similar manner and over time, sharing high amounts of intimate information likely reciprocally builds more trust and closeness (Altman & Taylor, 1973). In mentoring relationships, intimate disclosures may be evident in sharing vulnerable emotional states (e.g. grief and loss) or topics like sex and sexuality, which become increasingly relevant during adolescence. Mentors’ intimate disclosure may signal that they trust their mentee, but could be overwhelming for early adolescents (see Liang et al., 2008). By contrast, low intimacy disclosure may communicate to mentees that mentors are not attempting or desiring to build closeness and trust. Moreover, disclosure amount and intimacy may combine (i.e., statistically interact) to predict mentee’s relationship quality. Social Penetration Theory posits that as dyads engage in progressively more intimate disclosures, they are also able to increase the amount of disclosure at similar or lower levels of intimacy (Altman & Taylor, 1973). In context, this could mean that high amount combined with high intimacy will be associated with the highest levels of mentee relationship quality.

Openness is a third distinct feature of disclosure. Actors regulate their privacy in part by adjusting their disclosure boundaries and the degree to which they are willing to disclose to partners (Petronio, 2002). Openness is distinct from amount of disclosure: an actor may disclose little, but when they do, eagerly share personal information. An actor who is less open could disclose a lot, but with a guarded or half-hearted demeanor. Openness in disclosure may show that the relationship is a safe space for sharing personal information with one another (Shier et al., 2020), while a lack thereof could stifle the development of their mentoring relationship (Lester et al., 2019). On this basis, there was an expectation that greater mentor openness would be associated with greater mentees’ relationship quality. There were no a priori expectations regarding interactions between openness and other dimensions of disclosure because openness—unlike amount and intimacy—is not related to the content of disclosure, but rather describes the tone and demeanor of the actor in the disclosure interaction.

#### Partner effects of mentees disclosing to mentors

Literature on mentee self-disclosure is scarce. Examples of mentees as actors of disclosure typically occur in the context of examining how mentors and programs should respond to mentees disclosing harm to mentors (Rhodes et al., 2009). Little is known about how mentee disclosure is associated with mentors’ relationship evaluations. It is plausible that mentee disclosure has beneficial effects on mentor relationship quality (Partner Effect B in Fig. 1) as disclosure does for social partners in general. In this case, the amount and intimacy of mentee disclosure could signal mentee’s trust of mentors as well as feelings of closeness and safety in the mentoring relationship, thereby affirming the positive relationship for mentors (Altman & Taylor, 1973). One recent qualitative study included remarks from mentors that low mentee openness hindered relationship development (Lester et al., 2019). Accordingly, greater amount, and particularly greater intimacy and openness, of mentee disclosure may be associated with mentors’ higher relationship quality.

#### Actor effects for mentors and mentees

As noted earlier, disclosure research is typically partner-focused, emphasizing how actor disclosure predicts partner relationship quality. However, actors’ disclosure may also be associated with their own evaluations of relationship quality. Examples of actor effects in non-mentoring contexts include findings that individuals whose friends listened to them when they needed to talk (i.e., had positive disclosure experiences) valued their friendships more (Fehr, 2004). Similarly, within romantic relationships, actors’ disclosure is positively associated with actors’ relationship quality when partners are responsive and disclose in return (Laurenceau et al., 1988). However, these actor effects appear to be dependent on the responses of close relationship partners rather than actors’ disclosure itself. In a prior study, mentors’ self-reported disclosure (combining amount and intimacy) was not associated with their own ratings of relationship quality (Dutton et al., 2022). For mentees, it is possible that greater intimacy and openness is positively associated with mentee relationship quality as indicative of trust in the mentor, but it is also possible that greater mentee disclosure intimacy and openness leaves mentees feeling vulnerable. Accordingly, no actor effects for mentors (see Actor Effect A, Fig. 1) or mentees (Actor Effect B, Fig. 1) were anticipated in this study.

In sum, the dyadic processes of self-disclosure described here recognize that mentor and mentee disclosure can have important effects on the discloser and their social partner (see Fig. 1). The complexity and fluidity of social interactions like disclosure is also evident, showing that capturing these dyadic processes requires the use of appropriate methods. By applying these techniques to youth mentoring research, the study described here, though small, provides preliminary evidence for how greater uptake of these methods could advance understandings of mentoring relationships.

## The Current Study

Because youth mentoring is a relationship-based intervention, a deeper understanding of communication processes that can facilitate mentor-mentee bonds is critical. While early studies have signaled a positive disclosure-relationship quality association when mentors disclose to mentees, it is also apparent the methods used in previous studies provide limited insights into how mentor and mentee evaluations of relationship quality are affected by dyadic disclosure interactions. This rationale informed two research aims for the study. The first aim was to use behavioral observation methods to illustrate the usefulness of such methods for understanding youth mentoring relationships. To do this, mentor-mentee dyads were video-recorded engaging in a discussion that was coded for the amount, intimacy, and openness of self-disclosure exhibited by both mentors and mentees during the dyadic interaction. The second aim was to investigate the partner and actor effects of mentors’ and mentees’ amount, intimacy, and openness of self-disclosure on relationship quality (see Fig. 1). For partner effects of mentor disclosure, more intimate mentor disclosure was expected to predict higher mentee relationship quality, while an interaction effect with amount of disclosure was also tested with the expectation that high amounts of low intimate disclosure would have negative repercussions for mentee’s relationship quality. Additionally, the possibility that greater mentor openness was associated with greater relationship quality was examined. Finally, actor effects of mentors’ disclosure, and partner and actor effects of mentees’ disclosure, were also tested, but due to the lack of relevant theory and research in the literature, strong predictions were not advanced.

## Methods

### Participants

The sample comprised of 49 youth mentoring dyads. Most dyads were female same-gender matches (69.4%), 26.5% were male same-gender matches, and 4.1% were different-gender matches. Mentors’ primary ethnic identification were NZ European (49%), Pasifika (14.3%), Māori (10.2%), Other European (10.2%), Asian (8.2%), and Other (8.1%). Mentees’ primary ethnic identification were Pasifika (51%), Māori (18.4%), Asian (14.3%), NZ European (10.2%) and Other/Unknown (6.1%). One-fifth of mentors (20.4%) and almost half of mentees (42.9%) identified with multiple ethnic backgrounds. Most dyads were cross-cultural (79.6%). Mentor age ranged from 19 to 59 years (M = 36.16, SD = 11.49) and from 12 to 19 for mentees (M = 16.16, SD = 1.50). Relationship length ranged from three to 25 months (M = 8.49; SD = 5.57).

Participants came from nine local youth programs in Auckland, New Zealand, a diverse urban area. Eight programs were community-based, while one was a school-based mentoring program. Programs varied in the purpose and approach to mentoring: three were focused on educational achievement and the transition from high school to university, two focused on leadership skills, one each focused on creative arts and life skills, and the remaining two delivered mentoring as part of general youth development support. Mentoring programs were initially approached via email with a brief on the study and a request to attend a program event (e.g., training or information evening) to advertise the study and collect contact information from interested pairs. Through this strategy, 46 dyads from six programs joined the study. Further recruitment occurred through social media (two pairs) and a youth mentoring conference (one pair). In addition to being part of a formal mentoring program, all pairs met two other study eligibility criteria: 1) a minimum relationship length criterion of three months to ensure all participating dyads had an established relationship to increase the likelihood the observed interactions were representative of how they interact under normal conditions; and 2) a mentee age criteria of 12–18 years, although one participant turned 19 between signing up for the study and then attending the research session.

### Procedures

#### Data collection

The study used an approach to data collection and coding based on observational methods used to assess processes in romantic relationships (Overall et al., 2009). Each laboratory-based observation session was comprised of two surveys and video-recorded mentor-mentee interactions during three activities. Participants first completed survey questions about themselves and their mentoring relationship prior to the observed activities. Immediately following the observed activities, participants completed survey questions about their experience during the session. The current study only uses data from the first survey. Participants completed both surveys electronically, with mentors and mentees in separate rooms accompanied by a researcher to provide instruction and answer questions. A card game began the video-recorded portion of the session, acting as an icebreaker for participants to become comfortable with the laboratory space. This was followed by a creative presentation activity, to observe collaboration and help seeking under pressure. The third activity was an emotion discussion designed to elicit self-disclosure and observe communication. Analysis for the current study used data from the third activity where the emphasis was on providing space for dyads to communicate personal experiences and feelings, reflecting the closeness and intimacy in their relationship. Participants were given seven cards, each with an emotion and matching emoji on it (excited, stressed, hurt/sad/upset, anger/frustration, happy, embarrassed, proud) and asked to discuss what these emotions mean to them and/or a time they experienced one or more of these emotions. Dyads could talk about as few or as many of the emotions as they wished, and in any order they wanted. The discussion task lasted for seven minutes. In total, sessions lasted approximately 1.5 h.

All participants 16 and over provided informed consent. Parental consent and participant assent were collected from mentees under 16. The University of Auckland Human Ethics Committee approved the ethical protocols for the study.

#### Data coding

Data coding was conducted by cultural informants (see Coan & Gottman, 2007) in that the two coders (first two authors) had relevant mentoring relationship expertise and understanding to contextualize the interactions. The coding process followed the approach used in a previous observational study of self-disclosure (Tan et al., 2012). Pair ID’s were randomized, and then coders watched each video twice, with each viewing focused on coding one person in the dyad following an A-B-B-A format (i.e., mentor then mentee for video one, mentee then mentor for video two, and so on) to control for order effects. Immediately after watching each video, coders independently rated the target participant on the disclosure variables (described in the Measures section below). Coders then shared and discussed their original ratings before providing finalized participant ratings, which could be adjusted based on the post-viewing coder discussion. During the post-observation discussions, coders shared insights with each other and debated discrepancies. The intention of this process was not to ensure coders ratings matched, but provided an opportunity to contextualize ratings and draw on each coder’s observation and expertise. For instance, discussions sometimes provided clarity when participants spoke quietly or mumbled, or helped connect pieces of a narrative across multiple disclosures during the interaction. When the two coders had rated all the pairs, they re-coded the first six pairs to ensure coding consistency across the sample. Interrater reliability of the coders’ independent pre-discussion ratings ranged from 0.746 to 0.897, and the final ratings following coder discussion ranged from 0.968 to 0.984.

### Measures

#### Self-disclosure

Coders rated mentors’ and mentees’ amount, intimacy and openness of disclosure using a Likert scale between 1 (low) and 7 (high) based on a holistic assessment of each dimension across the full seven minute recorded interaction. Ratings of amount captured how much the mentor/mentee revealed information about themselves to their partner. Coders considered both the number of incidences of self-disclosure as well as the amount of information revealed in each instance of disclosure. Ratings of intimacy assessed how much the discloser revealed personal or intimate facts about themselves. Intimacy was rated bearing in mind the context of the interactions occurring within a mentoring relationship between an adult and young person, rather than the type and level of intimacy in other types of relationships (e.g., between adults or within a family). Finally, ratings of openness assessed how much the discloser appeared to be genuinely trying to open up and share themselves with their partner (see Online Resource 1 for full coding schedule).

#### Relationship quality

Mentors and mentees completed the same relationship quality measure, which involved rating their relationship on a 7-point scale (1 = not at all, 7 = extremely) for six dimensions associated with their relational bond: closeness, commitment, enjoyment, liking, satisfaction, and trust (e.g., How satisfied/happy are you with your mentoring relationship?; Dutton et al., 2022). Reliability of the relationship quality scale was high for both mentors (Cronbach’s α = 0.823) and mentees (Cronbach’s α = 0.935).

### Analysis

Analysis was conducted using an APIM approach to examine the effects of mentors’ and mentees’ self-disclosure on relationship quality (Kenny et al., 2006). This approach allows tests of the associations between one person’s behavior and their own outcomes (actor effects) and their interaction partner’s outcomes (partner effects) while accounting for the shared associations in outcomes across dyads. As shown in Fig. 1, in the context of this study, actor effects for mentors refer to the associations between mentors own self-disclosure behavior and mentors’ own relationship quality, whereas partner effects refer to the associations between mentors own self-disclosure behavior and mentees’ relationship quality. Thus, applying APIM models allowed testing of whether the mentors’ amount, intimacy, and openness of disclosure were associated with mentees’ relationship quality (partner effects) while accounting for any associations between mentors’ self-disclosure behavior and their own relationship quality (actor effects) as well as any shared associations between mentors’ and mentees’ relationship quality. Tests for the same associations but with mentees and mentors in opposite roles were also part of the APIM model, examining links between mentees’ own self-disclosure and mentees’ own relationship quality (actor effects), and partner effects between mentees’ own self-disclosure behavior and mentors’ relationship quality.

Analysis procedures followed the guidelines and syntax provided by Kenny et al. (2006) to run APIM models for distinguishable dyads using the MIXED procedure in SPSS 26. These models treat mentor and mentee scores from the same dyad as repeated measures and account for nonindependence within each dyad by modeling a compound symmetry error structure (Kenny et al., 2006). Given the levels, meaning, and effects of disclosures are likely to be invariant across mentors and mentees, two-intercept models were conducted to simultaneously estimate parameters for mentors and mentees while accounting for dependencies across dyad members. Shared associations across disclosure behavior were controlled for by modeling amount, intimacy and openness simultaneously. This allowed simultaneous estimates of each path shown in Fig. 1 to calculate the unique actor and partner effects of mentor and mentee disclosure amount, intimacy, and openness on relationship quality. All predictor variables were grand-mean centered.

## Results

Descriptive analyses show on average, disclosure amount was higher among mentees (M = 5.09, SD = 1.32) compared to mentors (M = 4.01, SD = 1.51). Mentee disclosure was also more intimate (M = 3.83, SD = 1.78) than mentors (M = 3.33; SD = 1.37), and mentees expressed greater openness (M = 5.41, SD = 1.49) compared to mentors (M = 4.91, SD = 1.54). Self-reported relationship quality was high for both mentees (M = 6.11, SD = 0.912) and mentors (M = 6.10, SD = 0.602). Bivariate correlations (Table 1) revealed a significant positive correlation between mentee openness and mentor relationship quality. However, these associations do not appropriately model the dependence across disclosure within and across mentors and mentees to isolate the partner and actor effects of self-disclosure, especially when dimensions are inevitably confounded, including correlations between amount, intimacy, and openness that may have opposing or interactive effects.

**Table 1:** Bivariate correlations between self-disclosure dimensions and relationship quality

Variables	1.	2.	3.	4.	5.	6.	7.
1. Mentor amount	–						
2. Mentor intimacy	0.285*	–					
3. Mentor openness	0.724**	0.567**	–				
4. Mentee amount	−0.170	0.136	0.020	–			
5. Mentee intimacy	0.109	0.838**	0.403**	0.351*	–		
6. Mentee openness	0.118	0.452**	0.340*	0.757**	0.566**	–	
7. Mentor relationship quality	−0.079	−0.029	0.128	0.132	0.006	0.301*	–
8. Mentee relationship quality	−0.196	0.092	−0.005	0.262	−0.015	0.227	0.159

^*^p < 0.05; **p < 0.01

The primary analyses modeled the main actor and partner effects displayed in Fig. 1. Mentee and mentor relationship quality was simultaneously regressed onto their own amount, intimacy and openness of disclosure (actor effects) and their partners’ amount, intimacy and openness of disclosure (partner effects). Table 2 displays the resulting actor and partner effects. Focusing first on mentee relationship quality (see left column of Table 2), a significant actor effect (shown in bold) indicated that greater intimate disclosure exhibited by mentees was associated with mentees reporting lower relationship quality. Controlling for these associations, two significant partner effects emerged (shown in bold in Table 2). Greater amount of disclosure displayed by mentors during the dyadic interactions was associated with mentees’ reporting lower relationship quality. By contrast, greater intimacy of disclosure exhibited by mentors was associated with mentees’ reporting higher relationship quality. Focusing on mentor relationship quality, only one significant partner effect emerged (see right column of Table 2). Greater openness of disclosure exhibited by mentees was associated with mentors’ reporting higher relationship quality. In sum, the results in Table 2 provide new evidence that partners’ self-disclosure is associated with both mentees’ and mentors’ relationship quality. Mentees’ evaluated their relationship more positively when mentors exhibited less overall amount, but highly intimate disclosure, whereas mentors evaluated their relationship more positively when mentees were more open.

**Table 2:** Actor and partner effects of amount, intimacy and openness of disclosure on mentees’ and mentors’ relationship quality

*Predictors*	*Mentee relationship quality*					*Mentor relationship quality*				
	*B*	*95% CI*	*t*	*p*	*r*	*B*	*95% CI*	*t*	*p*	*r*
Actor effect of amount of disclosure	0.15	−0.12, 0.43	1.12	0.264	0.12	−0.18	−0.39, 0.03	−1.74	0.086	0.19
Actor effect of intimacy of disclosure	−**0.38**	−**0.62**, −**0.14**	−**3.14**	**0.002**	**0.32**	−0.15	−0.47, 0.18	−0.89	0.374	0.10
Actor effect of openness of disclosure	0.12	−0.15, 0.38	0.88	0.380	0.10	0.18	−0.06, 0.41	1.51	0.136	0.16
*Partner* effect of amount of disclosure	−**0.24**	−**0.44**, −**0.02**	−**2.21**	**0.030**	**0.23**	−0.20	−0.47, 0.08	−1.43	0.156	0.15
*Partner* effect of intimacy of disclosure	**0.41**	**0.09, 0.74**	**2.54**	**0.013**	**0.27**	−0.04	−0.28, 0.20	−0.33	0.740	0.04
*Partner* effect of openness of disclosure	0.09	−0.14, 0.32	0.77	0.444	0.08	**0.30**	**0.04, 0.56**	**2.27**	**0.026**	**0.24**

When predicting *mentee’s* relationship quality, partner effects represent the associations between *mentors’* disclosure and mentees’ relationship quality (Partner Effect A, Fig. 1), whereas actor effects represent the associations between mentees’ disclosure and mentees’ relationship quality (Actor Effect B, Fig. 1). When predicting *mentor’s* relationship quality, partner effects represent the associations between *mentees’* disclosure and mentors’ relationship quality (Partner Effect B, Fig. 1), and actor effects represent the associations between mentors’ disclosure and mentors’ relationship quality (Actor Effect A, Fig. 1). Significant effects are shown in bold. *r* = Approximated effect size

**Fig. 2:**
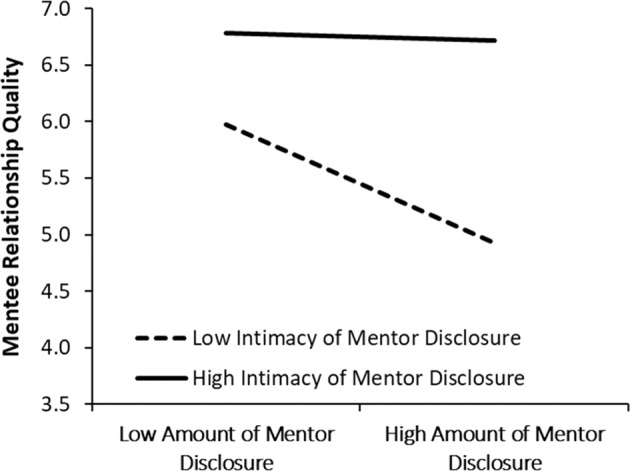
Significant interaction between amount and intimacy of mentor disclosure on mentee relationship quality

The secondary analyses examined whether the disclosure variables combined to predict relationship quality. For example, considering the two significant partner effects of mentor disclosure on mentee relationship quality, an expected interaction between amount and intimacy of disclosure could reveal that mentees’ evaluate their relationship more negatively when they are paired with mentors who display high amounts of disclosure with lower intimacy. To test this possible combination, primary analyses were rerun with actors’ amount X intimacy interaction and partners’ amount X intimacy interaction added into the model. One significant interaction emerged (shown in bold in Table 3). As shown in Fig. 2, greater amount of disclosure displayed by mentors was associated with lower mentee relationship quality when mentors’ disclosure was lower in intimacy (dashed line: *B* = −0.35, 95% CI [0.11, 0.71], *t* = −2.97, *p* = 0.005), but not when mentors’ disclosure was high in intimacy (solid line: *B* = −0.02, 95% CI [−0.27, 0.31], *t* = −0.14, *p* = 0.890). Equivalent models testing the amount X openness and intimacy X openness interactions were also run. No significant interactions emerged in predicting mentee or mentor relationship quality.

**Table 3:** Actor and partner effects of amount, intimacy and openness of disclosure, and amount x intimacy interactions, on mentees’ and mentors’ relationship quality

*Predictors*	*Mentee relationship quality*					*Mentor relationship quality*				
	*B*	*95% CI*	*t*	*p*	*r*	*B*	*95% CI*	*t*	*p*	*r*
Actor effect of amount of disclosure	0.11	−0.16, 0.39	0.83	0.410	0.09	−0.18	−0.40, 0.03	−1.68	0.096	0.19
Actor effect of intimacy of disclosure	−**0.35**	−**0.60**, −**0.09**	−**2.74**	**0.008**	**0.29**	−0.14	−0.47, 0.19	−0.86	0.393	0.10
Actor effect of openness of disclosure	0.15	−0.11, 0.42	1.17	0.247	0.13	0.18	−0.05, 0.42	1.54	0.157	0.17
Actor effect of amount X intimacy of disclosure	−0.04	−0.15, 0.07	−0.74	0.460	0.08	0.01	−0.10, 0.12	0.18	0.858	0.02
*Partner* effect of amount of disclosure	−0.18	−0.40, 0.03	−1.71	0.091	0.19	−0.20	−0.47, 0.08	−1.44	0.153	0.16
*Partner* effect of intimacy of disclosure	**0.47**	**0.15, 0.80**	**2.88**	**0.005**	**0.31**	−0.02	−0.27, 0.23	−0.15	0.882	0.02
*Partner* effect of openness of disclosure	0.13	−0.11, 0.36	1.08	0.285	0.12	**0.30**	**0.04, 0.57**	**2.26**	**0.026**	**0.25**
*Partner* effect of amount X intimacy of disclosure	**0.12**	**0.01, 0.23**	**2.12**	**0.037**	**0.23**	−0.03	−0.14, 0.08	−0.54	0.591	0.06

When predicting *mentee’s* relationship quality, partner effects represent the associations between *mentors’* disclosure and mentees’ relationship quality (Partner Effect A, Fig. 1), whereas actor effects represent the associations between mentees’ disclosure and mentees’ relationship quality (Actor Effect B, Fig. 1). When predicting *mentor’s* relationship quality, partner effects represent the associations between *mentees’* disclosure and mentors’ relationship quality (Partner Effect B, Fig. 1), and actor effects represent the associations between mentors’ disclosure and mentors’ relationship quality (Actor Effect A, Fig. 1). Significant effects are shown in bold. *r* = Approximated effect size

## Discussion

In a relationship-based field like youth mentoring, examining relational processes is essential to advancing research and practice that ultimately supports mentors and mentees to develop and sustain quality relationships (Varga & Deutsch, 2016). However, a reliance on retrospective self-report methods that are not suited to capture the dyadic, multidimensional, and dynamic nature of interpersonal communication has stifled emerging knowledge in this area (Pryce et al., 2021). This study offers an illustrative example of how behavioral observation and dyadic modeling can provide new insights into the mentor-mentee communication patterns that are associated with higher versus lower quality mentoring relationships. These methods provided a direct view of mentor-mentee disclosure interactions minimizing self-report biases and method variance and enabled assessment of how both mentors’ and mentees’ disclosure was associated with each other’s relationship quality. Behavioral observation also allowed objective, standardized measurement of different disclosure dimensions that are theorized to have differential effects. Theoretical and empirical work on self-disclosure suggests that disclosure in mentor-mentee dyads might operate in both similar and different ways to other types of close relationships. The current study was particularly concerned with understanding how the effects of self-disclosure might manifest differently depending on whether it is the mentors or the mentees who are disclosing (see Fig. 1). Analysis of these data in combination with mentor and mentee self-report measures of relationship quality using APIM revealed novel, though preliminary, insights about the dynamic effects of disclosure within a dyadic youth mentoring context.

### Partner Effects of Mentors Disclosing to Mentees

The APIM analyses highlighted the impact of mentors’ disclosure on mentees in particular. As expected, more intimate mentor disclosure was associated with higher mentee relationship quality, which aligns with theories of self-disclosure that clearly situate intimate disclosure as a primary mechanism for promoting close and trusting relationships (Altman & Taylor, 1973). The importance of intimate disclosures was reinforced by the interaction between mentor disclosure amount and intimacy. Mentors’ high amounts of disclosure actually predicted lower ratings of relationship quality for mentees, but only when mentors disclosed a large amount of non-intimate information. Mentees may interpret large amounts of low intimacy disclosures from their mentors as mentors dominating interpersonal communication without indicating signs of trust or offering opportunities for closeness. Excessive disclosure at a surface level may also leave less room for mentees to be active partners in the relationship and create distance between mentor and mentee (Dutton et al., 2020). Scholars examining disclosure in youth work relationships have cautioned similarly, noting that youth worker disclosure should focus on empathy and empowerment for youth, which excessive disclosure will inhibit (Murphy & Ord, 2013).

Previous research has suggested there may be a ‘tipping point’ for disclosure in youth-adult relationships, whereby a positive linear association between disclosure and relationship quality begins to decrease once disclosure amount reaches some threshold (Dutton et al., 2022). The negative association found in this study does not suggest mentors should refrain from disclosing in general; rather, a lot of meaningless disclosure about themselves to simply fill in gaps in conversation might have a detrimental impact on their relational connection. On the other hand, mentors who avoid disclosure in favor of turning conversations back to mentees may inadvertently signal that they do not want a genuinely mutual relationship with their mentee (Spiekermann et al., 2021). Mentors who practice attunement in these interactions are likely to notice mentee cues and pick up when and how to disclose in a manner that is supportive of their mentees’ needs (Pryce, 2012).

Returning to the importance of sharing intimate disclosures, it seems likely that mentees interpret intimate mentor disclosure as a positive indication about how their mentor feels about them, a sign their mentor is committed to the relationship and sees the mentee as an important person in their life (Liang et al., 2008). The positive relationship between mentors’ intimate disclosure and mentee ratings of relationship quality may also signal that mentors are able to disclose intimately in ways that are appropriate for the relationship and respect mentee boundaries, without overwhelming or emotionally burdening mentees with disclosure. Nevertheless, finding the right balance of intimacy is critically important for mentor disclosure. The context of a youth-adult relationship necessitates caution when interpreting the finding of high intimacy disclosures on the part of mentors being positive for youth mentoring relationships. In the interactions observed in this study, disclosures rated as highly intimate often involved sharing emotional states or reflecting on relationships with family and other loved ones. Such disclosures gave mentees insight into the inner workings of their mentor and it was evident mentors were disclosing to show their mentee a different side of them. The observational data did not show mentors discussing intimate topics, such as drug and alcohol use, although previous studies have indicated these conversations do occur (Dutton et al., 2019). Despite fostering closeness in the relationship, inappropriately disclosing intimate information (e.g. past engagement in risk behaviors) could indirectly reinforce such behaviors in a young mentee. Moreover, disclosing emotional states can also be inappropriate if it requires mentees to respond in ways they are uncomfortable or unprepared for (e.g., consoling a mentor who is visibly upset). Ultimately, mentors are responsible for ensuring their disclosures are benefiting the mentoring relationship and do not place undue emotional burden on their mentees. This reflection on the current results indicates that mentors need to be supported to differentiate between intimate disclosure that can be used in beneficial ways for developing and sustaining the mentoring relationship whilst limiting any potential iatrogenic effects (Dutton et al., 2022). Once again, mentor attunement is likely a critical skill for finding this balance (Pryce, 2012).

### Partner Effects of Mentees Disclosing to Mentors

One partner effect was found between mentees’ disclosure and mentors’ relationship quality. Greater mentee openness was associated with high mentor relationship quality. Whereas amount and intimacy focus on characteristics of the information that is disclosed, mentee openness represents a more general approach to disclosure that signals a genuine, active attempt to open up and share with mentors. Thus, mentors evaluated their relationship more positively when mentees showed a desire for their mentor to genuinely know and understand them. This suggests mentors can interpret disclosure-based signals other than content as evidence that the relationship is doing well. Even if mentees do not disclose a lot, or do not disclose very intimate information, mentee openness appears to provide assurance to mentors that mentees are willing to engage with them in the mentoring relationships. This finding is important because encouraging mentors to prioritize mentee openness during disclosure may reduce pressure on mentees to disclose in ways they may not be comfortable with, which relates to the potential dangers of mentee disclosure.

### Actor Effects of Disclosure

The sole actor effect that emerged illustrates the complexities of intimate disclosure in mentoring relationships, and the value of using dyadic methods to understand relationship processes. Although mentors’ more intimate disclosure was associated with higher mentee relationship quality (partner effect), mentees’ own intimate disclosure was associated with lower mentee relationship quality (actor effect). These contrasting associations are somewhat at odds with theoretical models of disclosure as a reciprocal process where both partners progressively increase the intimacy of their disclosures, which deepens and solidifies the relationship. Yet, there are some plausible reasons for this pattern to occur in youth mentoring dyads in particular. First, the youth-adult dynamic is embedded in distinct power relations and developmental processes (Keller & Pryce, 2010). These conditions may mean mentees are not comfortable with sharing intimate personal information with an adult, even if they like receiving it. As a developmental task of adolescence, mentees may still be negotiating their own sense of privacy and boundaries as it pertains to disclosure (Petronio, 2010). If mentors pressure mentees to disclose information they do not want to, this could have a detrimental effect on the relationship. Second, it is possible that mentees have had negative experiences disclosing to their mentor. Negative mentor responses to mentee disclosure—for instance, ignoring, not taking them seriously, or responding in an invalidating manner—could result in mentees having a pessimistic view of the mentor and their mentoring relationship. It may also create regret and anxiety about disclosing personal information. Recent research in natural mentoring relationships showed mentees who experienced negative responses to self-disclosure refrained from future disclosure (Rivens et al., 2021). Instead, mentees reported appreciation for mentor responses including active listening, understanding youth perspectives, and being non-judgmental (Rivens et al., 2021). It is clear that mentors need to be supported through training and ongoing supervision to consistently respond to mentee disclosures in such relationship-building ways.

### Implications and Caveats

Taken together, the present findings suggest self-disclosure and mentee perceptions of mentoring relationships are connected in complex and noteworthy ways. Mentees seem to be sensitive to disclosure, whether it comes from their mentor or themselves, and the presence of negative associations raises particular concerns about how disclosure manifests in mentoring relationships (Dutton et al., 2022). Like mentoring practice in general, self-disclosure needs to be mentee-oriented. This may present a challenge for mentors who struggle to adapt typical characteristics of disclosure within adult relationships, like reciprocity, to suit the needs of mentees. Crucially, there is scant research about self-disclosure from the perspective of mentees but their voice is essential for contextualizing what is known about disclosure in mentoring relationships, and then applying that knowledge to practice.

Talking is the most common activity that mentors and mentees do together (Herrera, 2004). As such, communication skills are commonly included in mentor training (Kupersmidt & Rhodes, 2014). Based on mentor reports, disclosure is likely to constitute a substantive portion of that talking time (Dutton et al., 2019). The presence of positive and negative associations between disclosure and relationship quality indicate a need for programs to be conscientious about how they train and prepare mentors to manage self-disclosure interactions. Programs should distinguish between training for day-to-day disclosures (e.g., about hobbies, families, school, and emotions) as they pertain to relationship development, and disclosures of harm that require program attention and intervention.

There are study limitations that temper the interpretation of the findings described here. As noted earlier, the small sample size of 49 dyads means the findings presented here provide only preliminary evidence of theoretically-relevant effects due to the limited statistical power to draw out effects. Replication at a larger scale is needed for investigating self-disclosure and other relational processes relevant to youth mentoring. One of the drawbacks of laboratory-based behavioral observation research is the time-intensive nature and cost (Pryce et al., 2021), which certainly impacted the scope of this project. This also limited the analyses to a cross-sectional design, despite the intention to conduct longitudinal analyses. The original study design included a follow-up questionnaire after six months, but low retention yielded a sample size too small for robust analysis. Research on self-disclosure in mentoring would benefit from a longitudinal investigation given existing theory and research in other contexts regarding how disclosure changes in terms of content, amount, and intimacy, through the course of a relationship (e.g., Willems et al., 2020). Longitudinal analyses would also help ascertain whether the directionality of the relationship between self-disclosure and relationship quality established in the disclosure literature is valid in the mentoring context. Similarly, relationship length was not included these analyses, although it was part of the eligibility criteria for the study. Future research could draw on models of relationship development in mentoring (e.g., Keller, 2005) to consider how relationship length and self-disclosure interact, particularly the progression of intimacy in disclosure.

Despite these caveats, this small-scale study demonstrates the substantial advantages behavioral observation paradigms along with APIM analyses offer in terms of dissecting the complexities of interdependent communication behaviors in youth mentoring relationships. The findings also have applicability to other forms of youth-adult partnerships where communication processes and associated effects are likely to be similar (e.g. relationships with professional youth workers or youth development program facilitators). Additional behavioral observation research on communication behaviors across a range of youth-adult partnerships would contribute importantly to training for adults working in professional and paraprofessional helping roles with young people, as well as the development of more targeted, evidence-informed interventions, as seen in research on other close relationships (see Heyman, 2001).

## Conclusion

Self-disclosure is a communication tool for building and deepening interpersonal relationships that is not well understood in the youth mentoring context. This study used behavioral observations of mentor-mentee interactions to examine whether self-disclosure differentially influences relationship quality depending on who is disclosing, and how. The findings showed some positive partner effects of disclosure: more intimate mentor disclosures predicted higher mentee relationship quality, and greater mentee openness predicted higher mentor relationship quality. Importantly, however, negative associations were also revealed: high amounts of low intimacy mentor disclosures and more intimate mentee disclosures predicted lower mentee relationship quality. These preliminary results are indicative of the unique impact disclosure has on mentees—whether it is their own or their mentor’s—and reinforces the fundamental orientation of mentoring practice that prioritizes mentee experiences and outcomes. The study also highlight the utility of applying well-established close relationship theories and methodologies to the mentoring context to draw out the idiosyncrasies of mentor-mentee relationships that must inform training and practice. Despite being a small study, it is illustrative of the potential knowledge contributions such methods offer and thus provide a foundation for larger-scale application of these methods. Youth mentoring interventions are popular during adolescence and continuing to build a robust evidence base of process-oriented research makes a valuable contribution towards enhancing the effectiveness of mentoring.

## Supplementary information

Supplementary Information
