# Supplementary material for: Using Observational Dyadic Methods in Youth Mentoring Research: Preliminary Evidence of the Role of Actors’ and Partners’ Self-disclosure in Predicting Relationship Quality
Source: J Youth Adolesc. 2023 Mar 4;52(6):1157–69. doi: 10.1007/s10964-023-01757-y (PMC10121698; doi:10.1007/s10964-023-01757-y)
Supplement: Supplementary file 1 — Supplementary Information [file 10964_2023_1757_MOESM1_ESM.docx]

**Electronic Supplementary Material (ESM 1)**

Dutton, H., Deane, K. L., & Overall., N. C. “Using Observational Dyadic Methods in Youth Mentoring Research: Preliminary Evidence of the Role of Actors’ and Partners’ Self-disclosure in Predicting Relationship Quality”, Journal of Youth & Adolescence.

Corresponding Author: Hilary Dutton, Faculty of Education, University of Canterbury, Private Bag 4800, Christchurch, New Zealand. Phone +643 369 0128. Email [hilary.dutton@canterbury.ac.nz](mailto:hilary.dutton@canterbury.ac.nz).

**Coding schedule for self-disclosure in youth mentoring relationships**

This coding schedule is designed to guide independent coders in assessing self-disclosure in an emotions discussion between mentors and mentees. Three dimensions of self-disclosure will be assessed using this coding schedule: Amount, Intimacy, and Openness.

**Self-disclosure**

Self-disclosure generally includes any way individuals reveal information about themselves to another person. This includes, but is not limited to, information about their experiences in the past or future (e.g., their experiences at school), their feelings or dispositions (e.g., “I’m an anxious person”), their context (e.g., details about their family or pet), and perceptions of the mentee or mentoring relationship (e.g., “I think we’ve grown together over this relationship’).

It is important to distinguish self-disclosure from general feedback, advice, or information sharing. Consider two comments from a mentor:

“If you’re stressed, maybe meditating or spending time relaxing with friends and family would help”.

“To deal with stress, I find it helpful to spend time relaxing with friends and family, or I might meditate”.

The first comment is feedback rather than disclosure because it does not reveal anything about the mentor. In the second comment, the same information is shared but reveals something personal about the mentor.

**Dimensions of mentor self-disclosure**

**Amount of Self-Disclosure:** Consider how much the mentor self-disclosed to their mentee and revealed information about themselves, including

- personal states (e.g., mood, thoughts, feelings),
- dispositions (e.g., type of person they are, skills or habits, identity labels),
- interests (e.g., media they enjoy, hobbies they participate in)
- values and beliefs (e.g., religious beliefs)
- personal behaviours (e.g., substance use)
- events in the past or future (e.g., aspirations or dreams for the future; positive or negative experiences at school or work).

Consider not only the number of incidences of self-disclosure, but the amount of information revealed in those disclosures, and whether the discloser took few/some/all of the opportunities for disclosure that were available to them in the discussion.

**Intimacy of Self-Disclosure:** Self-disclosure can be meaningful, deep, self-revealing and personal (intimate) versus superficial, impersonal and revealing little about the thoughts, feelings and qualities of the individual (non-intimate).When rating the intimacy of self-disclosure, consider the extent to which personal or intimate facts are revealed about the self and whether the information provided illuminates the type of person the individual is. Also consider the context of the emotion discussion as *an interaction within a youth mentoring relationship between an adult and young person*—intimacy in this context might not look the same as it does in other types of relationships (e.g., between adults or within a family).

Indicators of high intimacy disclosures may include:

- Content: disclosures of intimate topics such as substance use or sex and relationships, or topics which express vulnerability and heightened emotions, such as grief or fears.
- Tone: disclosures which provoke an emotional response in the participant (e.g., teariness).
- Participant expressing difficulty with talking about the disclosure (e.g., “this is a hard thing to me to say”, “this isn’t something I like to talk about much”).

Indicators of low intimacy disclosures may include:

- Content: disclosures on superficial topics, such as hobbies/interests, positive aspects of school and work.
- Tone: disclosures which are given in a matter of fact, impersonal way.

**Openness of discloser:** Openness refers to the extent to which it appears the discloser is genuinely trying to open up and share themselves with their partner. Unlike the other dimensions, which describe the disclosures, openness describes the demeanour of the discloser: it is a personal trait, rather than a characteristic of the disclosure interaction.

Disclosers who are open may share themselves in a wholehearted way, and put themselves in their disclosure in a way that allows their partner to really see who they are. A discloser low in openness may engage in disclosure that is guarded, half-hearted, or disingenuous.

**Coding**

Ratings will be provided on 7-pt scales to *globally* capture the degree to which each individual exhibits high, moderate, or low levels for each dimension. Coders will watch the entire interaction and take into account the frequency, intensity, and duration of behaviours associated with each dimension (i.e., low = 1-2, moderate = 3-5, high = 6-7). Coders will watch each video twice, with each viewing focused on coding one person in the dyad following an A-B-B-A format (i.e., mentor then mentee for video one, mentee then mentor for video two, and so on).

Mentoring self-disclosure coding sheet Coder: ______________

Emotional discussion Date: _______________

Pair ID: ____________

|  | **Low** | | | | | | **High** | **Final** |
| --- | --- | --- | --- | --- | --- | --- | --- | --- |
| **Amount** of disclosure | 1 | 2 | 3 | 4 | 5 | 6 | 7 |  |
| **Intimacy** of disclosure | 1 | 2 | 3 | 4 | 5 | 6 | 7 |  |
| **Openness** of discloser | 1 | 2 | 3 | 4 | 5 | 6 | 7 |  |

Pair ID: ____________

|  | **Low** | | | | | | **High** | **Final** |
| --- | --- | --- | --- | --- | --- | --- | --- | --- |
| **Amount** of disclosure | 1 | 2 | 3 | 4 | 5 | 6 | 7 |  |
| **Intimacy** of disclosure | 1 | 2 | 3 | 4 | 5 | 6 | 7 |  |
| **Openness** of discloser | 1 | 2 | 3 | 4 | 5 | 6 | 7 |  |

Pair ID: ____________

|  | **Low** | | | | | | **High** | **Final** |
| --- | --- | --- | --- | --- | --- | --- | --- | --- |
| **Amount** of disclosure | 1 | 2 | 3 | 4 | 5 | 6 | 7 |  |
| **Intimacy** of disclosure | 1 | 2 | 3 | 4 | 5 | 6 | 7 |  |
| **Openness** of discloser | 1 | 2 | 3 | 4 | 5 | 6 | 7 |  |

Pair ID: ____________

|  | **Low** | | | | | | **High** | **Final** |
| --- | --- | --- | --- | --- | --- | --- | --- | --- |
| **Amount** of disclosure | 1 | 2 | 3 | 4 | 5 | 6 | 7 |  |
| **Intimacy** of disclosure | 1 | 2 | 3 | 4 | 5 | 6 | 7 |  |
| **Openness** of discloser | 1 | 2 | 3 | 4 | 5 | 6 | 7 |  |
